# Sevoflurane depletes macrophages from the melanoma microenvironment

**DOI:** 10.1371/journal.pone.0233789

**Published:** 2020-05-29

**Authors:** Isabella Sztwiertnia, Judith Schenz, Katharina Bomans, Dominik Schaack, Johanna Ohnesorge, Sandra Tamulyte, Markus A. Weigand, Florian Uhle

**Affiliations:** 1 Department of Anesthesiology, Heidelberg University Hospital, Heidelberg, Germany; 2 Department of Anesthesiology and Intensive Care Medicine, University Hospital Tübingen, Eberhard-Karls-University Tübingen, Tübingen, Germany; Universidade de Sao Paulo, BRAZIL

## Abstract

**Background:**

With more than 18 million annual new cases, cancer belongs to the major challenges of modern healthcare. Surgical resection of solid tumours under general anaesthesia is the prime therapy. Different aspects of anaesthesia are under discussion to independently influence the long-term outcome of cancer patients. Most recently, the commonly used volatile anaesthetics like sevoflurane have entered the spotlight, as retrospective studies suggest a detrimental outcome in certain cancer aetiologies with sparse mechanistic understanding. Our objective was to investigate this concept in a murine melanoma model, herein comparing the consequence of inhalative and injection anesthesia on tumour composition and growth.

**Methods:**

We used a murine model of malignant melanoma in male, adult C57BL/6 mice (n = 92), induced by the subcutaneous injection of B16-F10 cells. We either exposed the melanoma cells to sevoflurane before implantation or subjected the animals to single or double anaesthesia with either volatile or injection drugs. After a maximum follow-up of 4 weeks, leucocytes within the tumour microenvironment (TME) were comprehensively analysed by flow cytometry with focus on tumor-associated macrophages (TAM).

**Results:**

We found that exposure of melanoma cells to sevoflurane before implantation induced long-lasting transcriptome changes and aggravated tumour growth, without extensive changes of the TME. Contrastingly, both a single and double anaesthesia with sevoflurane led to a significant reduction of TAMs (injection vs. sevoflurane: 2,0 vs. 0.3% and 1.2 vs. 0.6%, respectively), whilst increasing PD-L1 expression on the remaining cells (mean fluorescent intensity injection vs. sevoflurane: 3,804 vs. 7,143 and 9,090 vs. 32,228, respectively). No changes in tumour growth were observed in these groups.

**Conclusion:**

In sharp contrast to the detrimental impact of sevoflurane on patients’ outcome reported in retrospective clinical studies, we propose here that sevoflurane might actually exert a beneficial effect by decreasing TAMs within the TME, rendering the tumour again susceptible for cytotoxic T cells and immunotherapies. Further research is warranted to delineate, how these results translate into the clinic.

## Introduction

According to WHOs *Global Cancer Observatory*, an estimate of 18.1 million new cases of cancer have occurred in 2018 worldwide [1]. In recent years, ground-breaking progress has been made in the treatment of previously difficult-to-treat cancers as, e.g. malignant melanoma [2], founded in the emergence of therapeutic “biologicals” like monoclonal antibodies and genetically-modified immune cells [3,4]. Especially the treatment of advanced melanoma was dramatically improved by these host-directed therapies, as melanoma resembles a cancer entity of high aggressivity and metastatic potential, not lastly due to its potent immune escape mechanisms [5,6]. Nonetheless, the surgical resection of neoplastic tissue is still a prime intervention for solid tumours, alongside chemo- and radiotherapy. General anaesthesia is nowadays an indispensable prerequisite of surgery and makes use of a plethora of different drugs to ensure the hallmarks hypnosis, analgesia, and relaxation. Several aspects of modern anaesthesia have been controversially debated for many years for their impact on cancer outcome–either by modulating the immune system or directly impacting the cancer cells [7]. The benefit of local and regional anaesthesia procedures has been extensively investigated without clear evidence [8]. The question about a detrimental impact of the commonly used volatile anaesthetics is arising, not least based on the striking success of tumour immunotherapy in the context of their proven immunomodulatory action [9]. Those discussions have been recently fuelled by the publication of a series of retrospective studies suggesting a worse outcome of patients after volatile anaesthesia compared to patients with total intravenous anaesthesia for certain cancers [10]. However, the mechanistic understanding behind the concept of “volatile harm” in cancer is sparse, currently relying broadly on more or less well conducted *in vitro* experiments on selected immune or cancer cells or observational studies on circulating immune cells [9], in particular lacking the crucial insights into the complex tumour immune microenvironment. Among several others, monocytes and macrophages are long-known to belong to the negatively affected cells by volatile anaesthesia, leading to a reduced inflammatory cytokine secretion and adhesion molecule expression upon exposure [11,12]. In the last decade, tumour-associated macrophages (TAMs) within the microenvironment gained increasing attention as potential therapeutic targets after understanding that they–in sharp contrast to the importance of their tissue counterparts in innate host response–actually serve the tumour, promoting its survival, proliferation, neo-angiogenesis, and even dissemination [13]. In line with this, a higher density of TAMs has been reported to be associated with poor prognosis in different cancer entities [14,15]. Once recruited to the tumour microenvironment, macrophages are reprogrammed and act as immune suppressors, actively shielding the tumour from cytotoxic T cells via expression of immune checkpoint ligands as, e.g., PD-L1 [16]. Therefore, the removal of TAMs from the microenvironment is even considered as a therapeutic target to break the resistance of certain tumours against checkpoint inhibitors [17,18].

We hypothesised that balanced anaesthesia with the broadly used compound sevoflurane might have the potential to reshape the immune tumour microenvironment in the B16-F10 induced murine standard model of malignant melanoma, known for its PD-L1-mediated immune escape mechanisms. Our study aims to examine this concept and to give insights into potential mechanisms underlying the recent epidemiological findings.

## Material and methods

### Animals

The project was approved and permission granted from the governmental animal welfare committee (File number G-237/17, Regierungspraesidium Karlsruhe). All experiments were conducted in accordance to national and international regulations for animal welfare. In total, 92 male C57BL/6J mice between 10–12 weeks of age were used for all experiments (Janvier Labs, Le Genest-Saint-Isle, France). Animals were housed under 12h light-dark cycle and constant temperature/humidity within a barrier animal facility in type II cages (maximum group size 4 animals) with wood chips bedding and enrichment with nesting material. Animals had free access to food and water over the whole experiment.

### Cultivation and implantation of melanoma cells

Murine skin melanoma cell line B16-F10 (ATCC no. CRL-6475) was obtained from the national standard repository (LGC Standards, Wesel, Germany). Full freedom of the cell line from the major 26 rodent-pathogenic viruses was ensured before experimental conduct using PCR diagnostics (Charles River Laboratories, Wilmington, USA). Cells were subconfluently cultivated in Dulbecco's Modified Eagle's Medium (DMEM) (Thermo Fisher Scientific, Waltham, USA) supplemented with 10% ultra-low endotoxin fetal bovine serum (ULE-FBS, Cell Concepts, Umkirch, Germany) under 37ºC and 5% CO_2_. For sub-cultivation, medium was removed and cells were enzymatically detached with 0.25% trypsin-EDTA (Thermo Fisher Scientific, Waltham, USA) before splitting.

For standardization, for each experimental group, a new stock of cryo-conserved B16-F10 cells was thawed and maintained for exactly one week (two passages) before injection. Cells were harvested on the experimental day as described for sub-cultivation, but resuspended into sterile PBS (Thermo Fisher Scientific, Waltham, USA) after several washing steps to remove excessive trypsin. Cells were held on ice until final injection. For tumour induction, animal’s flank was shaved, locally disinfected using 70% ethanol, and 1x10^5^ B16-F10 cells (in 100μl PBS) were injected subcutaneously (s.c.) using a 27 gauge needle. Proper injection was affirmed by bleb formation.

Priming of B16-F10 cells with sevoflurane was accomplished within a closed hypoxia chamber located in an 37ºC heating cabinet. Cell flasks were put into the chamber and the chamber was flushed with 2% sevoflurane (Baxter, Unterschleissheim, Germany) in normal air with 21% O_2_ for 30min, followed by an incubation for additional 2h. Subsequently, cells were prepared and injected into the animals within 2h. Processing of the cells before injection was as described above.

### Study design

**Fig 1:**
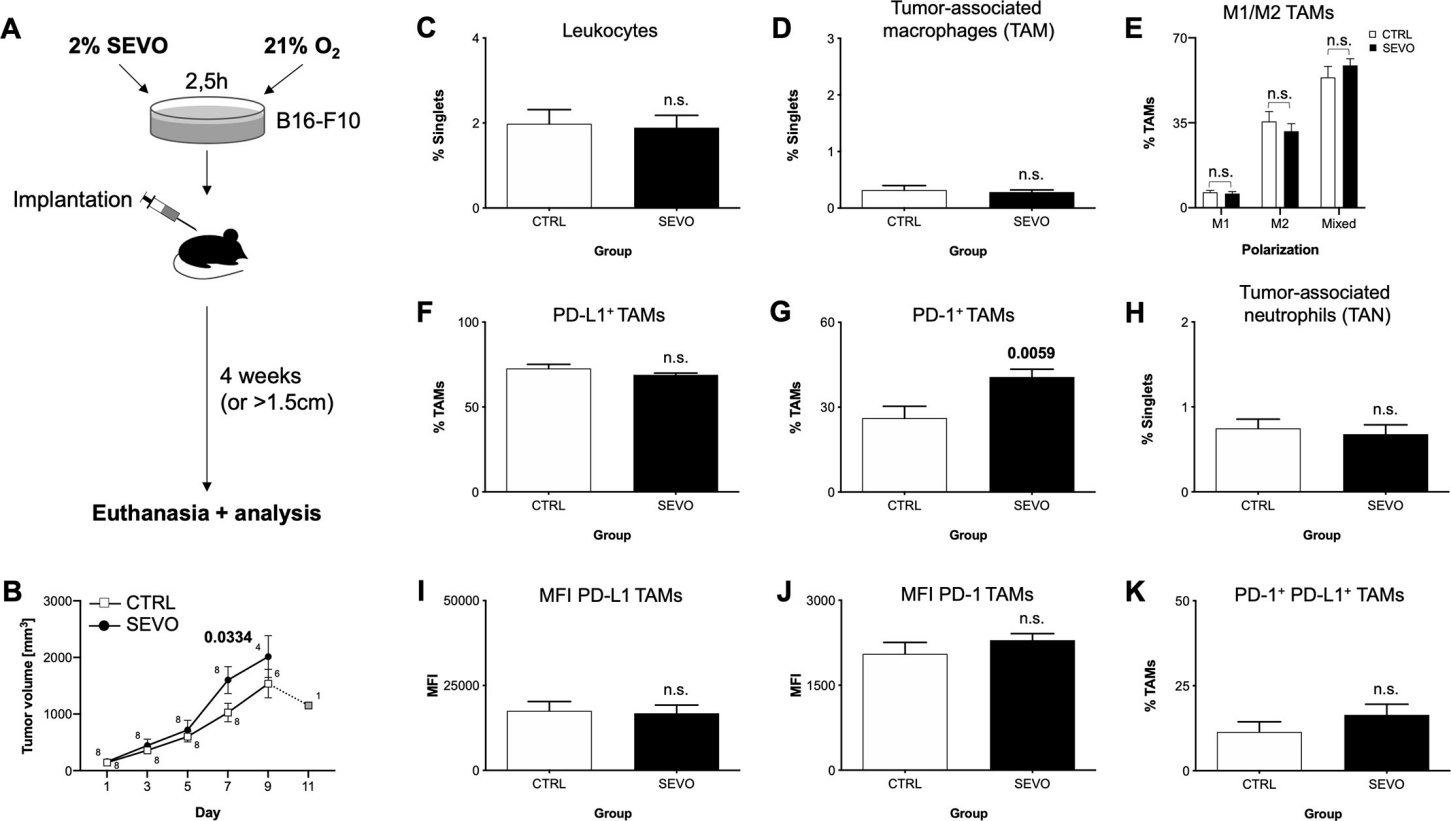
Impact of sevoflurane priming of B16-F10 cells on tumour microenvironment. (A) B16-F10 melanoma cells were incubated for 2,5h with 2% sevoflurane and injected after 24h. (B) Tumour volume development between the groups. Small numbers indicate group size on each timepoint. Grey box indicates only one animal remaining in the control group.(C) Percentage of CD45^+^ leucocytes, (D) Tumour-associated macrophages (TAMs), (E) distribution of TAM polarization into M1, M2 or mixed phenotype, (F) PD-L1^+^ TAMs, (G) PD-1^+^ TAMs, (H) TANs, (I) MFI of TAM PD-L1, (J) MFI of TAM PD-1, (K) PD-1^+^ PD-L1^+^ TAMs. SEVO: sevoflurane group (black bars), CTRL: control (white bars), PD-L1: Programmed death-ligand 1, PD-1: Programmed death 1, TAN: tumour-associated neutrophils, MFI: mean fluorescence intensity, n.s.: not significant. Bars represent mean and standard error of mean. Bold numbers indicate significant differences (*P-*value <0.05) between groups (n = 8 animals each group), calculated with either t-test or Mann-Whitney-U test.

Animals received either no anaesthesia during implantation of sevoflurane primed cells (Fig 1A, “primed”), a single cycle of anaesthesia (1h) on the day of implantation (Fig 3A, “single”), or in total two cycles of anaesthesia (1h each) with one on the day of implantation as well as one 7 days later (Fig 4A, “double”). Each anaesthesia cycle was timed for exactly 1h from start of induction to antagonization or stop of sevoflurane exposure, respectively. For animals receiving no anaesthesia, the primed cells were s.c. injected into the awake animal while fixed by a second experimenter. For the anaesthesia groups, cells were injected 5min before the end of the first anaesthesia cycle. Animals were randomly grouped on the day of experiment.

Anaesthesia with sevoflurane (SEVO group) was induced by putting the animals into a cabinet flushed with 8% sevoflurane (100% O_2_). After loss of righting reflex, anaesthesia was maintained with 3–4% sevoflurane (100% O_2_) over a nasal cone for a total of 1h. In addition, the mice received s.c. injection of 5μg/kg fentanyl (Janssen-Cilag GmbH, Neuss, Germany). Temperature homeostasis was maintained using a heating plate below the animals.

Injection anaesthesia (INJ group) was induced by s.c. application of 5μg/kg fentanyl, 2mg/kg midazolam (Hameln Pharma Plus GmbH, Hameln, Germany), and 0.15mg/kg medetomidine (Orion Pharma GmbH, Hamburg, Germany). After injection, animals were located into a cabinet with 100% O_2_. After 1h, anaesthesia was partly antagonised by s.c. application of 0.2mg/kg flumazenil (Roche, Basel, Switzerland) and 0.75mg/kg atipamezol (Orion Pharma GmbH, Hamburg, Germany).

Follow-up assessment of the animals included body weight and caliper-based tumour size measurement every two days (or more frequent in case of large tumours). Tumour volume was calculated according to the formula (V_Tumor_ = (Width^2^ x Length)/2)). For analysis, day of first palpable tumour was set to “1”. After a maximum of 4 weeks after injection, animals were euthanised and the tumour was resected for further analysis. We experienced a 100% take-on rate over all groups. Euthanasia was performed by intraperitoneal application of 120mg/kg ketamine (Pfizer Deutschland GmbH, Berlin, Germany) and 16mg/kg xylazine (Bayer Vital GmbH, Leverkusen, Germany), followed by cardiac puncture and exsanguination. In case of a tumour dimension >1.5cm, a skin ulceration near the tumour or a loss of body weight >20%, the respective animal was immediately euthanised and subjected to further analysis.

A separate set of experiments were performed on animals without tumour injection. Animals were euthanised 24h after anaesthesia (as described above) and bone marrow was isolated for further flow cytometric analysis of cell composition as well as to evaluate monocyte function.

### Bone marrow monocyte isolation and stimulation

For bone marrow extraction, both intact femurs of the animals were rapidly extracted after euthanasia and muscle tissue was removed before further processing. Bone marrow cavity was opened under laminar flow on both sides and the bone was flushed several times with RPMI1640 medium (Thermo Fisher Scientific, Waltham, USA) using a 25G cannula. Cells were passed through a 70μm sieve before flow cytometry and cell isolation.

Monocytes were negatively isolated from bone marrow cells by depletion of non-target cells via labelling with antibodies linked to magnetic beads (Monocyte Isolation Kit (BM)) according to the manufacturer’s instructions using an automatised AutoMACS Pro system (both Miltenyi Biotech, Bergisch Gladbach, Germany). Purity of isolation was checked by flow cytometry and reached >90% CD11b^+^ Ly6C^+^ cells.

For in vitro stimulation, cells were resuspended into RPMI1640 supplemented with 10% FBS, placed into a 96-well microplate (50.000 monocytes/well), and stimulated with 100ng/ml ultrapure lipopolysaccharide (LPS, *E*.*coli* 0111:B4), 250μg/ml zymosan (Zymosan depleted) (both Invivogen, San Diego, USA) or mock for 24h. TNF-α and IL-6 supernatant levels were quantified by DuoSet ELISA (Bio-Teche, Minneapolis, USA).

### Tumour cell homogenization

Tumour mass was resected from animals after euthanasia. As a prerequisite for flow cytometry, tumour dissociation was realised by a combination of mechanical force and enzymatical digestion (Tumor Dissociation Kit, mouse, Miltenyi Biotech, Bergisch Gladbach, Germany), using a gentleMACS™ Octo Dissociator with Heater (program: 37C_m_TDK_1) at 37ºC according to the manufacturer’s instructions.

### Flow cytometry

For characterization of the tumour-infiltrating myeloid cells, 1x10^6^ cells of the tumour suspension were stained (15min, 4ºC) with the following antibodies.: LIVE/DEAD™ Fixable Violet Dead Cell Stain (Thermo Fisher Scientific, Waltham, USA), CD45-APC/Cy7 (Cat.No. 103115, Clone 30-F11), F4/80-PerCP/Cy5.5 (Cat.No. 123127, Clone BM8), CD206-PE (Cat.No. 141705, Clone C068C2) (all from Biolegend, San Diego, USA), CD11b-FITC (Cat.No. 561688, Clone M1/70), Ly-6G-PE/Cy7 (Cat.No. 560601, Clone 1A8), MHCII-Alexa647 (Cat.No. 562367, Clone M5/114.15.2), PD-1-PE (Cat.No. 561788, Clone J43), and PD-L1-APC (Cat.No. 564715, Clone MIH5) (all from BD Bioscience, Heidelberg, Germany). Cells were washed three times with PBS with 0.5% BSA (Carl Roth, Karlsruhe, Germany) before measurement. Leukocytes were characterised as CD45^+^ singlets, tumour-associated macrophages (TAMs) as CD45^+^/CD11b^+^/F4/80^+^, tumour-associated neutrophils (TANs) as CD45^+^/CD11b^+^/Ly-6G^+^. Full gating strategy is provided in S1 Fig. Macrophages were further classified according to their positivity for MHCII (M1), CD206 (M2), or as mixed (MHCII^+^/CD206^+^). Tumour cells were characterised as CD45^-^ singlets. Expression of PD-1 and PD-L1 on TAMs/tumour cells was assessed as percentage of positive singlets as well as mean fluorescence intensity (MFI). For all three proteins, proper gating was achieved by using fluorescence-minus-one (FMO) control tubes.

For analysis of bone marrow composition, 1x10^6^ cells were stained (15min, 4ºC) with the following antibodies: Lineage-Pacific Blue (Cat.No. 133305, Clones 17A2/ RB6-8C5/ RA3-6B2/ Ter-119/ M1/70), Sca-1-PE/Cy7 (Cat.No. 122513, Clone E13-161.7), c-Kit-APC (Cat.No. 135107, Clone ACK2), CD48-APC/Cy7 (Cat.No. 103431, Clone HM48-1), CD150-PerCP/Cy5.5 (Cat.No. 115921, Clone TC15-12F12.2) (all from Biolegend, San Diego, USA), CD34-FITC (Cat.No. 130-105-890, Clone REA383), and CD16/32-PE (Cat.No. 130-107-065, Clone REA377) (both from Miltenyi Biotech, Bergisch Gladbach, Germany). Cells were washed three times with PBS with 0.5% BSA (Carl Roth, Karlsruhe, Germany) and fixed with 4% formaldehyde. After fixation, another washing step was performed before data acquisition. FMO control tubes were used to facilitate proper gating of CD34, CD16/32, and CD150. Full gating is depicted in S2 Fig, and cell populations were phenotypically defined as follows: Hematopoietic stem cells (HSC; Lin^-^/Sca-1^+^/c-Kit^+^), Long-term-HSC (LT-HSC; Lin^-^/Sca-1^+^/c-Kit^+^/CD48^-^/CD150^+^), Short-term-HSC (ST-HSC; Lin^-^/Sca-1^+^/c-Kit^+^/CD48^-^/CD150^-^), Common myeloid progenitor (CMP; Lin^-^/Sca-1^-^/c-Kit^+^/CD16/32^int^/CD34^+^), Megakaryocyte-erythroid progenitor (MEP; Lin^-^/Sca-1^-^/c-Kit^+^/CD16/32^int^/CD34^-^), Granulocyte-monocyte progenitor (GMP; Lin^-^/Sca-1^-^/c-Kit^+^/CD16/32^+^/CD34^+^).

Acquisition of cells was done on a FACSVerse flow cytometer, followed by data analysis using the FACSuite software (BD Bioscience, Heidelberg, Germany). All antibodies were individually titrated to reach best signal-to-noise ratio.

### RNA sequencing

For isolation of tumour RNA, small pieces of tissue were put into 1ml of TRIzol® reagent (Thermo Fisher Scientific, Waltham, USA) together with CK18 ceramic beads (1.4mm), followed by disruption in a Precellys® bead mill (Bertin Instruments, Montigny-le-Bretonneux, France) for up to 5 cycles of 20sec each with 5.000rpm. For RNA isolation, RNeasy Mini Kit (Qiagen, Hilden, Germany) was used. Purity and quantity were evaluated in a Nanodrop™ photometer (Thermo Fisher Scientific, Waltham, USA), integrity on a Bioanalyzer system with RNA 6000 Nano Kit (both Agilent Technologies, Santa Clara, USA). Next generation sequencing was performed as external service on an Illumina HiSeq 2500 platform (Eurofins Genomics Germany, Ebersberg, Germany) and all samples have been batch-processed throughout library preparation and sequencing to minimize technical bias. Raw data has been uploaded in the Gene Expression Omnibus (GEO) repository and is publicly available under the record number GSE135690.

### Bioinformatics

Raw RNA-seq datasets were subjected to initial quality control using FastQC. The subsequent data processing pipeline included a filtering step using SortMeRNA for removal of contaminating ribosomal RNA [19], and downstream trimming of short or low quality reads conducted by Trimmomatic software [20]. The remaining reads were passed to reference genome alignment by STAR using *Mus musculus* release M17 (GRCm38.p6) reference genome as readily available from GENCODE project (https://www.gencodegenes.org) [21]. Comprehensive gene annotation on the primary assembly (chromosomes and scaffolds) was chosen as superset of the main annotation. Unique and unambiguously mapped reads were selected for further analysis. Data conversion to sorted binary alignment format (BAM) was conducted using SAMtools [22]. The respective release M17 gene transfer file was used in conjunction with replicate BAM files for feature counting using HTSeq [23]. For downstream analysis of count data, Biocondutor DESeq2 package was used in R environment [24]. The resulting differentially expressed genes were filtered with thresholds of absolute linear fold change values at or above 1.5 and p-values below 0.02. In reference to the resulting lists of differentially expressed genes, over-represented GO-terms were identified with Genomatix Genome Analyzer (version 3.70808, Intrexon Bioinformatics Germany GmbH, Munich, Germany) for the entire list of resulting genes as well as separated gene sets of up- and down-regulated genes. GO-term bar plots display the top 10 over-represented GO-term results in respect of attributed p-values for the full set of dis-regulated genes and the sub-selection of up-regulated genes respectively. For heatmap generation, count data of library-size normalised differentially expressed genes were selected. Pre-processing included gene-wise calculation of z-score standardised values of normalised count data as well as gene- and sample-wise clustering based on Ward’s hierarchical agglomerative clustering method (Euclidean distance measure; Ward2 criterion).

### Statistical analysis

All statistical analysis and visualization were performed with Prism software (version 8.1.2, GraphPad Software, San Diego, USA). Pairwise group comparison of metric data was conducted using Student’s t test or Mann-Whitney U (one-tailed), depending on the results of the *a priori* performed Shapiro-Wilk test for normality. Differential survival analysis was performed using Log-rank test.

A *P*-value below 0.05 was anticipated as significant. All data is visualised as mean and standard error of mean (SEM).

## Results

### Sevoflurane priming modulates tumour transcriptome and growth

Our starting point was to test, if sevoflurane exposure of the melanoma cells alone (and not the whole organism) might introduce changes in growth and immune cell composition of the developing tumour *in vivo* (Fig 1A). In the group which received primed cells, we found a significant increase of tumour volume on day 7 after first occurrence (1026±163 vs. 1,601±239mm^3^, CTRL vs. SEVO, *p* = 0.0334) (Fig 1B). This was neither accompanied by differences in body weight development between the exposure groups (S3A Fig), nor with earlier tumour occurrence or euthanasia (S4A+S4D Fig). We further examined the tumour microenvironment regarding the presence of immune cells. No differences were found in the proportion of leucocytes (Fig 1C), TAMs (Fig 1D), and tumour-associated neutrophils (TANs) (Fig 1H), as well as the expression of PD-L1 (Fig 1F+1I), and markers of macrophage polarization (MCH-II and CD206) (Fig 1E). However, the proportion of PD-1^+^ TAMs was significant increased within the tumour (26.1±11.9 vs. 40,7±7.7%, CTRL vs. SEVO, *p* = 0.0059) (Fig 1G), without an accompanying increase in the antigen density (Fig 1J), indicating an expansion of this TAM subpopulation. Contrastingly, the tumour cells themselves did not exhibit alterations of PD-1/PD-L1 expression (S3 Fig).

**Fig 2:**
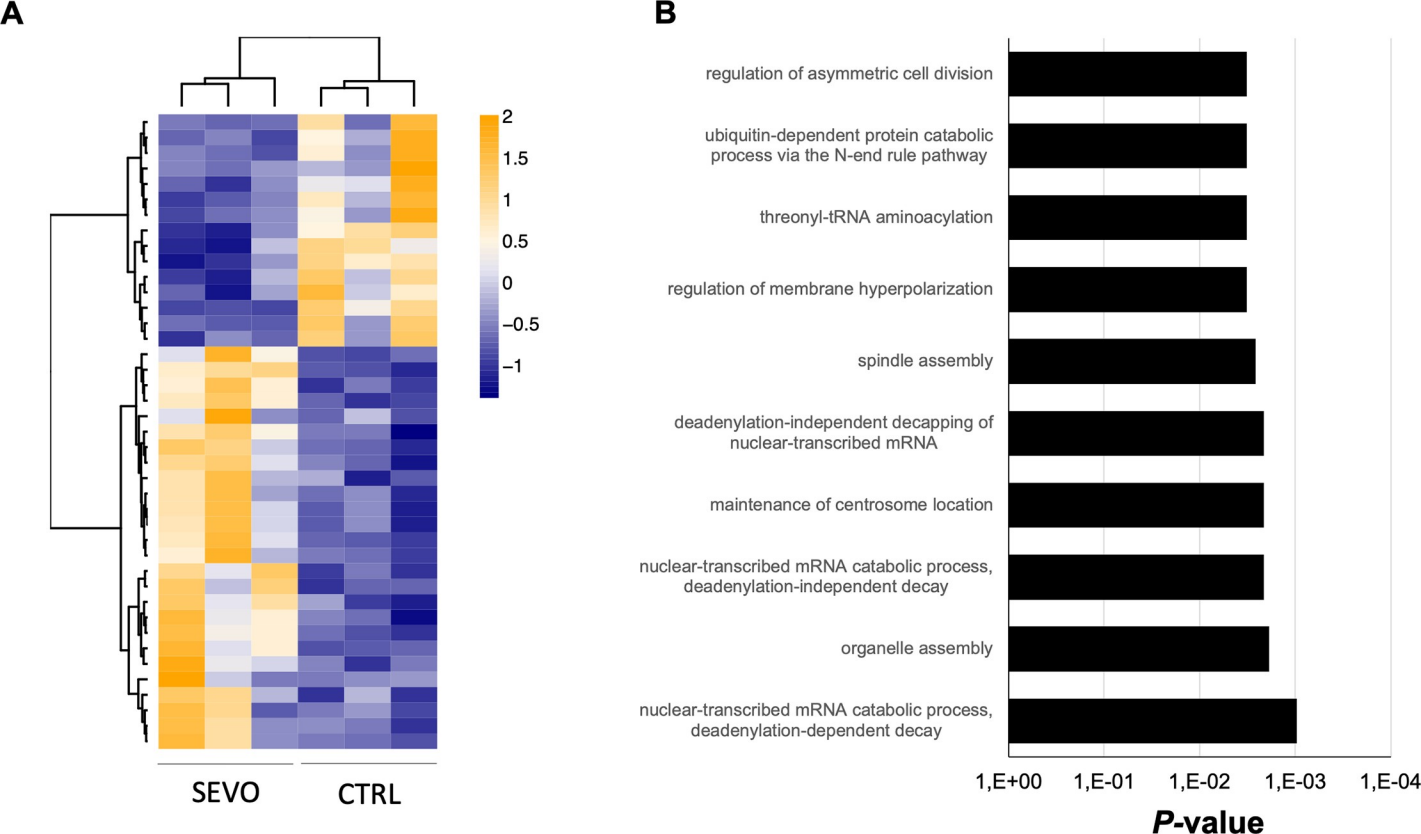
Tumour gene expression after sevoflurane priming and in vivo growth. (A) Heatmap representation of the 41 differentially expressed genes (26 genes up-, 15 genes down-regulated; linear fold change ≥1.5, *P*-value <0.02), comparing sevoflurane-primed samples to controls (n = 3 samples of each group). (B) Gene ontology analysis showing the first 10 overrepresented biological processes with *P-*value <0.01.

We therefore asked, if the observed changes in growth might be reasoned by transcriptomic changes, preserved from priming throughout tumour development. Using RNA-seq, we found subtle (41 genes in total), yet significant changes in the transcriptome of tumours arising from sevoflurane-primed cells (Fig 2A) (full gene list is provided in S1 Table). Strikingly, those genes were related to crucial biological processes for cell division like, e.g. “organelle assembly”, “maintenance of centrosome location” or “regulation of asymmetric cell division” (Fig 2B). In summary, we can prove that sevoflurane introduces changes in gene expression, which might project into faster proliferation and tumour growth, while changes of the immune milieu are reduced to more PD-1^+^ TAMs. However, this might hint towards a tumour-immune-crosstalk.

### Single anaesthesia with sevoflurane dampens macrophage recruitment and phenotype

**Fig 3:**
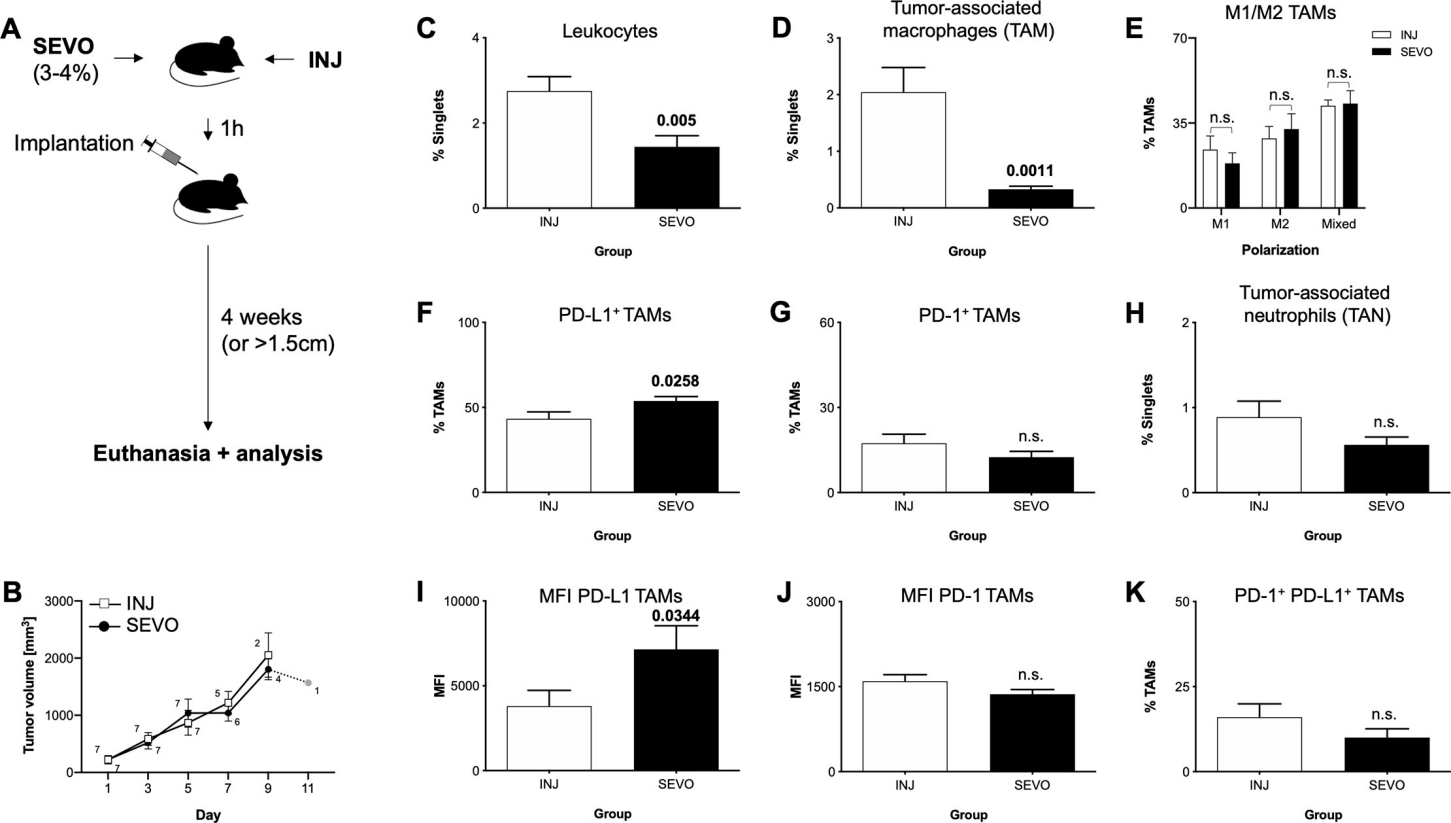
Impact of a single anesthesia on the melanoma tumour microenvironment. (A) Animals received a single cycle of anesthesia (1h) with either volatile (SEVO) or injection (INJ) anesthetics. Tumour cells were implanted at the end of anesthesia and animals followed up for a maximum of 4 weeks. (B) Tumour volume development between the groups. Small numbers indicate group size on each timepoint. Grey box indicates only one animal remaining in the INJ group.(C) Percentage of CD45^+^ leucocytes, (D) Tumour-associated macrophages (TAMs), (E) distribution of TAM polarization into M1, M2 or mixed phenotype, (F) PD-L1^+^ TAMs, (G) PD-1^+^ TAMs, (H) TANs, (I) MFI of TAM PD-L1, (J) MFI of TAM PD-1, (K) PD-1^+^ PD-L1^+^ TAMs. SEVO: sevoflurane group (black bars), INJ: control group with injection anesthesia (white bars), PD-L1: Programmed death-ligand 1, PD-1: Programmed death 1, TAN: tumour-associated neutrophils, MFI: mean fluorescence intensity, n.s.: not significant. Bars represent mean and standard error of mean. Bold numbers indicate significant differences (*P-*value<0.05) between groups (n = 7 animals each group), calculated with either t-test or Mann-Whitney-U test.

After clarifying the impact on tumour cells alone, we wanted to assess in the next step the impact of sevoflurane anaesthesia on the total organism during tumour development, including the immune system. Therefore, mice were subjected to anaesthesia (either sevoflurane or injection) for 1h before implantation of melanoma cells, thereby reducing the exposure of these cells to a minimum (Fig 3A). In contrast to the former results with primed melanoma cells, we found no differences in the development of the tumour volume between the anaesthesia groups (Fig 3B). Similarly, no differences in body weight development (S3B Fig), time to tumour occurrence or euthanasia were found between the groups (S4B+S4E Fig). Strikingly, we found changes in the tumour immune milieu: proportion of leucocytes was reduced (2.8±0.3% vs. 1.4±0.3%, INJ vs. SEVO, *p* = 0.005) (Fig 3C), founded mainly on a loss of TAMs (2.0±0.4% vs. 0.3±0.15%, INJ vs. SEVO, *p* = 0.0011) (Fig 3D), with no changes in TAN recruitment (Fig 3H). While polarization of the remaining macrophages was not altered (Fig 3E), both the proportion of PD-L1^+^ TAMs (43.2±4.1% vs. 53.8±2.6%, INJ vs. SEVO, *p* = 0.0258) as well as the antigen density of PD-L1 on TAMs (MFI: 3,804±927 vs. 7,143±1,391, INJ vs. SEVO, *p* = 0.0344) were increased after sevoflurane (Fig 3F+3I). No changes in PD-1 expression or double positive TAMs were found (Fig 3G+3J and 3K). The tumour cells showed no changes in the expression of PD-1 (S3A+S3C Fig), but the already PD-L1^+^ cells exhibited an increased expression of PD-L1 antigen (S5B+S5D Fig).

Based on these findings, we asked about the impact of anaesthesia on the function of naïve monocytes within the bone marrow. We isolated those from mice 24h after they were subjected to the same anaesthesia intervention as described before and stimulated them with either LPS or zymosan. Intriguingly, we found neither changes in TNF-α or IL-6 cytokine response (S6A+S6E Fig), nor in the composition of the hematopoietic niche at all (S6B–S6D, S6F–S6H Fig). Taken together, we can clearly show a impact of sevoflurane, but not anaesthesia in general, on the composition of the tumour immune milieu, while naïve monocytes are not affected.

### Double exposure to sevoflurane aggravates the immunomodulatory effect

**Fig 4:**
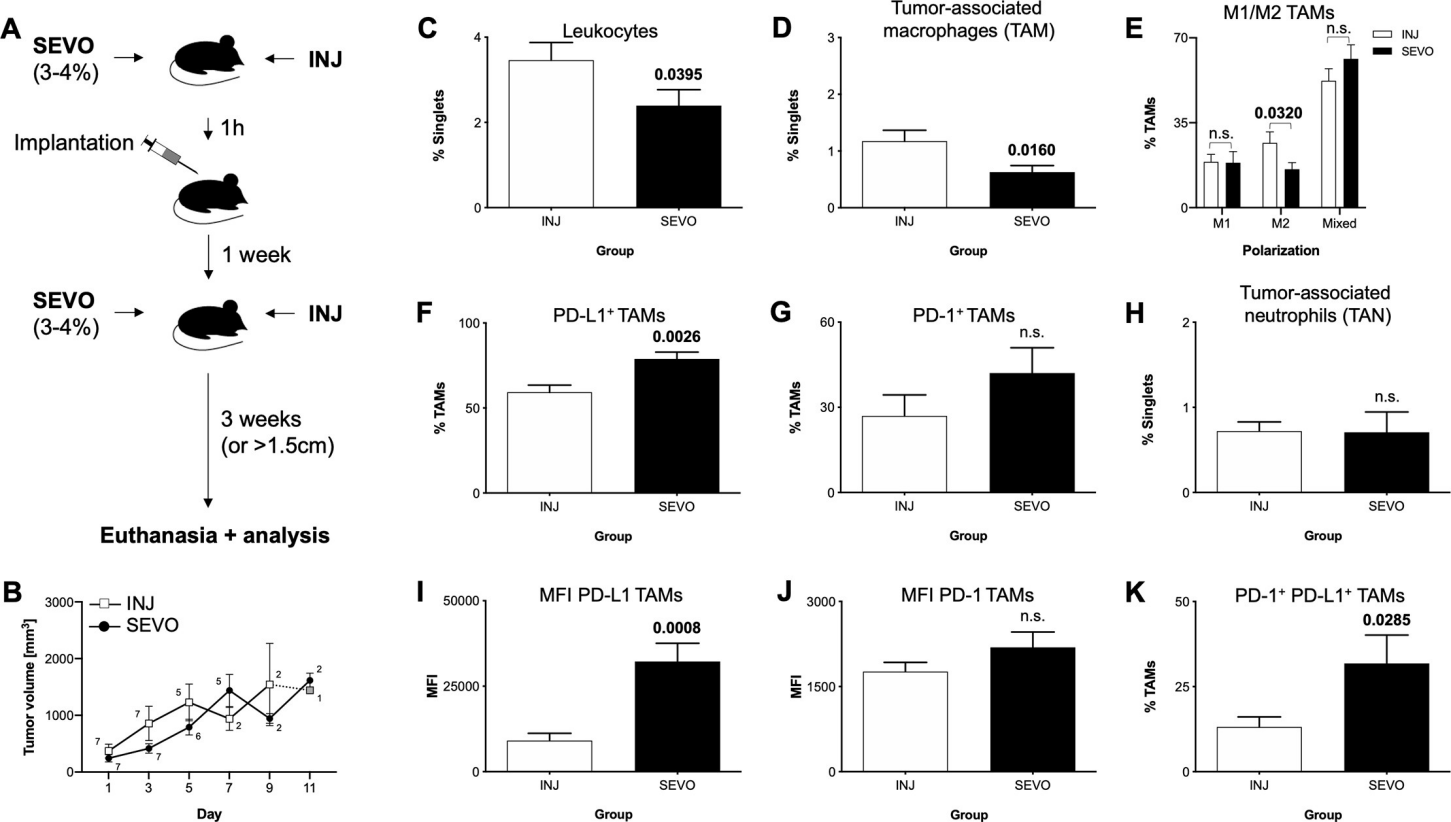
Impact of two sequential anesthesia on the melanoma tumour microenvironment. (A) Animals received a single cycle of anesthesia (1h) with either volatile (SEVO) or injection (INJ) anesthetics at time of tumour cell implantation, as well as a second cycle of the same anesthesia one week later. Animals were followed up for a maximum of 3 weeks afterwards. (B) Tumour volume development between the groups. Small numbers indicate group size on each timepoint. Grey circle indicates only one animal remaining in the SEVO group.(C) Percentage of CD45^+^ leucocytes, (D) Tumour-associated macrophages (TAMs), (E) distribution of TAM polarization into M1, M2 or mixed phenotype, (F) PD-L1^+^ TAMs, (G) PD-1^+^ TAMs, (H) TANs, (I) MFI of TAM PD-L1, (J) MFI of TAM PD-1, (K) PD-1^+^ PD-L1^+^ TAMs. SEVO: sevoflurane group (black bars), INJ: control group with injection anesthesia (white bars), PD-L1: Programmed death-ligand 1, PD-1: Programmed death 1, TAN: tumour-associated neutrophils, MFI: mean fluorescence intensity, n.s.: not significant. Bars represent mean and standard error of mean. Bold numbers indicate significant differences (*P-*value<0.05) between groups (n = 7 animals each group), calculated with either t-test or Mann-Whitney-U test.

Eager to merge the previous approaches and to come closer to clinical reality, we performed experiments with two sequential anaesthesia separated by one week. Tumour cells were implanted at the end of the first anaesthesia and were therefore not exposed to the anaesthesia, while the whole organism (including immune system) was. After one week, the animals received a second cycle of the same anaesthesia, thereby exposing both tumour cells as well as the organism to the anaesthetic agents (Fig 4A). As before, we found no differences in the development of the tumour volume (Fig 4B), in body weight development (S3C Fig), time to tumour occurrence or euthanasia between the anaesthesia groups (S4C+S4F Fig). In line with the results of the single anaesthesia approach, we observed lower leucocyte (3.5±0.4% vs. 2.4±0.4%, INJ vs. SEVO, *p* = 0.0395) and TAM infiltration (1.2±0.2% vs. 0.6±0.1%, INJ vs. SEVO, *p* = 0.016) into the tumour (Fig 4C and 4D), with a significant smaller CD206^+^ M2 subpopulation among the latter (26.7±4.5% vs. 15.8±2.8%, INJ vs. SEVO, *p* = 0.032). Infiltrating TANs remained unchanged (Fig 4H). Strikingly, the increase of PD-L1 found after single anaesthesia was reproduced in a pronounced fashion, with both PD-L1^+^ TAMs (59,3±4.1% vs. 78.9±4.0%, INJ vs. SEVO, *p* = 0.026) (Fig 4F), but especially the antigen density of PD-L1 on TAMs exhibiting a dramatic increase in the sevoflurane group (MFI: 9,090±2,123 vs. 32,228±5,335, INJ vs. SEVO, *p* = 0.0008) (Fig 4I). Contrasting, in the double anaesthesia approach also PD-1 trended towards an increase after sevoflurane, while yet not reaching statistical significance (Fig 4G and 4J). These mimics the alterations observed with primed melanoma cells and leads to an expansion of PD-1^+^ PD-L1^+^ double positive TAMs, which did not occur in the other experimental approaches (13.2±2.9% vs. 31.8±8.4%, INJ vs. SEVO, *p* = 0.0285) (Fig 4K). In conclusion, exposing the animals to repeated anaesthesia augments the alterations of macrophage immunity within the tumour microenvironment.

## Discussion

We report here the results of our *in vivo* study, using an established murine model of orthotopic melanoma, aiming to unravel the impact of general anaesthesia with the volatile agent sevoflurane on the tumour microenvironment (TME) in a controlled design. Our analysis focused on TAMs, due to their hypothesized importance in the tumour resistance to therapy and endogenous anti-tumour immunity. While the exposure of melanoma cells before implantation alters their proliferation and transcriptome after *in vivo* tumorigenesis, profound alterations of immune cells within the TME were only induced when the whole animals received an inhalative anaesthesia, resulting, e.g., in a substantial decrease of leucocytes, predominantly TAMs.

We first evaluated the influence of sevoflurane priming on melanoma cells and found an increased tumour growth in vivo and more PD-1^+^ TAMs within the TME. Intriguingly, this contradicts the recent findings of Meier and colleagues, which did not observe a size difference in a similar approach [25]. However, this group used isoflurane in their experiments, raising the question if the three nowadays commonly used compounds isoflurane, desflurane, and sevoflurane evoke the same effect. A solitary study exposed ovarian cancer cells to all three agents in comparable minimal alveolar concentrations (MAC) and found distinct expression profiles for genes involved in metastasis formation, hinting towards a compound-specific action [26]. If this is just a transient effect or it persists over time and impacts proliferation remains elusive, as they performed the analysis as early as 6h after exposure. In contrast, we used RNA-seq and approached the transcriptome after in vivo growth several weeks after exposure. The subtle alterations we found match the increased growth observed, however, they cannot explain the isolated increase of PD-1^+^ TAMs we found as well. We hypothesise this might be a secondary effect of accelerated tumour growth, with a higher number of tumour cells locally secreting more potent cytokines as G-CSF, leading to an attraction of monocytes and tissue macrophages to this location [27]. The observed phenotype might be the result of a tumour escape mechanism, as PD-1^+^ TAMs are susceptible to receive tumour cell-dependent PD-L1 signals, inhibiting the phagocytic capacity of the macrophages and thereby again promoting tumour growth [28].

Separating the influence of sevoflurane on melanoma cells and the immune system in our *in vivo* approach, expanding the experimental results of others, clearly shows that melanoma cells undergo alterations upon exposure to sevoflurane, projecting into larger tumours by reprogramming infiltrating TAMs. In the next step, we strived to unravel the isolated impact of sevoflurane on immune cells in the context of a developing tumour. The melanoma cells were therefore implanted at the end of anaesthesia. We observed a loss of leucocytes and especially TAMs from the TME, a phenomenon at first sight in agreement with the proposed immunosuppressive effect of sevoflurane on monocytes. However, some aspects need to be considered in this context: first, circulating monocytes possess a short lifespan in the blood of approximately one day in mice and human [29–31], implicating that anaesthesia-exposed circulating monocytes are long gone at the time the tumour starts to grow. Second, our results prove that sevoflurane does neither alter naïve monocyte responsivity, when isolated directly from the bone marrow 24h after anaesthesia, nor change the composition of the hematopoietic niche. Last, tissue macrophages under steady state do not solely originate from those circulating precursors, but are to a large extent seeded during embryonic development and are capable of proliferating and replenishing [32]. Even more intriguingly, these embryonic macrophages have been shown to expand and represent the main TAM fraction during the development of pancreatic ductal adenocarcinoma [33]. We hypothesize from this that macrophages from the nearby tissue around the injection site, rather than circulating monocytes. Further cell tracing approaches can clarify the fate of tumor macrophages during anesthesia, e.g. by using recently generated myeloid reporter systems [34].

The remnant TAMs within the tumour of mice after sevoflurane anaesthesia exhibit an increase in PD-L1 expression, resembling the phenotype of “classical” pro-tumorigenic macrophages, capable to block effective CD8+ T cell responses [16]. Also, TAM PD-L1 can deliver signals to the recently discovered subpopulation of PD-1^+^ melanoma cells and drive the proliferation [35]. We did not observe differences in tumour growth after single anaesthesia, which might be reasonable considering the small subpopulation of remaining TAMs after sevoflurane anaesthesia, which might not be sufficient to drive this proliferative effect. However, our results instead hint towards a surprising direction: although not affecting tumour growth, sevoflurane anaesthesia—compared to injection anaesthesia—depleted the TME of shielding TAMs, which might be considered a beneficial effect. The anti-tumour activity of CD8^+^ T cells might still be hampered through the high expression of PD-L1 by the B16F10 melanoma cells. Nevertheless, the loss of TAMs might be an important prerequisite for a successful response to immunotherapy with PD-L1:PD-1 blocking antibodies. Hoves and colleagues provided evidence a beneficial loss of TAMs by applying a combination therapy of inhibitory α-CSF-R and agonistic α-CD40 to melanoma mice. This treatment led to a hyperactivation and subsequent depletion of TAMs, rendering the tumours susceptible for immunotherapy [36]. Besides, TAMs have recently been shown to scavenge therapeutic PD-1 antibodies from infiltrating T cells in an Fc-dependent manner, thereby excerting an opposing action on the antibody therapy [37].

Of importance, the above results can be resembled when applying two cycles of anaesthesia to the animals, thereby exposing both the immune system as well as the melanoma cells as happening in clinical reality. Interestingly, the increase in TAM PD-L1 expression was stronger compared to a single exposure so sevoflurane. This might hint towards a synergetic interplay of the TME and sevoflurane exposure. One hallmark of the TME is low oxygen tension, due to the lack of supplying vasculature [38]. Hypoxia leads to the stabilization of the transcription factor HIF-1α and its translocation to the nucleus, where it induces expression of downstream target genes, including PD-L1 as well as genes of the glycolytic pathway, needed to maintain energy homeostasis in the absence of oxygen [39]. Apart from low oxygen, sevoflurane itself has been shown to induce HIF-1α in tumour cells [40].However, this increase of PD-L1 occurs simultaneously with a numerical depletion of TAMs from the TME.

Our study implies limitations, worth to be discussed. Above all, murine cancer models using cell line grafts might not fully mimic all hallmarks of carcinogenesis, e.g., angiogenesis. We decided to approach our hypothesis using the established and highly standardized B16-F10 model [41], however, this concept needs to be tested in other relevant models of melanoma and other solid tumours to prove the generalizability of our results. Aiming to come as close to clinical conditions as possible, we compared the effect of sevoflurane anaesthesia with injection anaesthesia. For the latter, we decided to change to mice-adapted anaesthesia, omitting propofol, which has substantial side effects in rodents. However, both regimes induced a stable anaesthesia state of surgical tolerance, removing the bias of other studies comparing anaesthesia to awake animals. Last, in our project, we focused on TAMs due to their proven importance, but would not exclude that other immune cells might be affected by sevoflurane as well. Regarding the sole use of sevoflurane, our choice was based on the fact that it is—together with desflurane–the standard compound for anesthesia worldwide [42]. Future work is necessary to delineate, if those two compounds exert differential effects on tumor and immune cells.

In conclusion, our results provide first evidence that sevoflurane, but not anaesthesia *per se*, exerts effects both on melanoma cells and immune cells, resulting in leucocyte changes within the TME. Most apparently, TAMs are decreased within the tumour after sevoflurane anaesthesia. This effect might open a gap for successful immunotherapy, however, further studies using the combination of anaesthesia and immunotherapies are critical to prove this idea. Our results might at first seem to contradict the findings of retrospective clinical studies, which report poor outcome for cancer surgery patients after volatile anaesthesia. However, we would rather suggest to consider it as a reminder of the tremendous tumour heterogeneity resulting from the origin and inherent genetical diversity, ultimately mounting into the individual response of each tumour to therapy. Based on our study and the result of others, it is time to reframe anaesthesia not only as a technical prerequisite of surgery, but rather as an integral part within the spectrum of cancer therapy, making its contribution for patients’ outcome. As a consequence, we further need to accept that anaesthesia needs to be adapted to the individual patient and tumour, setting the stage for “personalized onco-anaesthesia” in the future.

## Supporting information

S1 Fig Gating strategy for analysis of TAMs, TANs and tumor cells by flow cytometry. Gates were set according to fluorescence-minus-one (FMO) controls as indicated in the example (plots with “FMO” in headline).
(TIFF)

S2 Fig Gating strategy for analysis of bone marrow progenitor cells by flow cytometry. Gates were adjusted according to fluorescence-minus-one (FMO) controls as indicated in the example (plots with “FMO” in headline) where necessary. HSC: Hematopoietic stem cells; LT-HSC: Long-term-HSC; ST-HSC: Short-term-HSC; CMP: Common myeloid progenitor; MEP: Megakaryocyte-erythroid progenitor; GMP: Granulocyte-monocyte progenitor.
(TIFF)

S3 Fig Normalised body weight development of all animals over the first 14 days after tumour cell implantation. Mice receiving (A) implantation of primed tumour cells (n = 8 animals each group), (B) single anesthesia (n = 7 animals each group), or (C) double anesthesia (n = 7 animals each group). INJ: group receiving injection anesthesia (white boxes); CTRL: group receiving non-primed B16-F10 cell (white boxes); SEVO: groups receiving sevoflurane anesthesia or sevoflurane-primed B16-F10 cells, respectively (black circles).
(TIFF)

S4 Fig Kaplan-Maier curves of time to euthanasia (A-C) or time to palpable tumour (D-E). Animals received primed B16-F10 cells (A+D), a single anesthesia (B+E), or double anesthesia (C+F). Each group with n = 7 animals with exception of both subgroups of the priming experiment, which has n = 8 animals in each group. P-values represent results of Log-rank test. INJ: group receiving injection anesthesia; CTRL: group receiving non-primed B16-F10 cell; SEVO: groups receiving sevoflurane anesthesia or sevoflurane-primed B16-F10 cells, respectively.
(TIFF)

S5 Fig Expression of PD-1 (A+C) and PD-L1 (B+D) on tumour cells. Results are given either as fraction of positive singlet cells (A+B) or mean fluorescence intensity (MFI) (C+D). All experimental groups are combined within each graph, with “single and “double” indicating the number of anesthesia cycles received (n = 7 animals each group), and “primed” the group receiving primed B16-F10 cells or control cells, respectively (n = 8 animals each group). PD-L1: Programmed death-ligand 1, PD-1: Programmed death 1, TAN: tumour-associated neutrophils, MFI: mean fluorescence intensity, n.s.: not significant. Bars represent mean and standard error of mean, with white bars representing the control groups and black bars the sevoflurane groups. Bold number indicates significant difference (*P-*value<0.05) between groups, calculated with either t-test or Mann-Whitney-U test.
(TIFF)

S6 Fig Analysis of immune function 24h after anesthesia. (A) TNF-α concentration (measured by ELISA) in the supernatant of bone marrow monocytes after 24h stimulation with 100ng/ml LPS or 250μg/ml zymosan (or untreated as control). White bars represent results of animals receiving injection anesthesia, black bars represent results from animals receiving sevoflurane anesthesia. Percentage of (B) hematopoietic stem cells (HSC) (C) Short-term HSC, and (D) Long term HSC. (E) IL-6 concentration (measured by ELISA) in the supernatant of bone marrow monocytes after 24h stimulation with 100ng/ml LPS or 250μg/ml zymosan (or untreated as control). Percentage of (F) Common myeloid progenitor, (G) megakaryocyte-erythroid progenitor, and (H) granulocyte-macrophage progenitor. Bars represent mean and standard error of mean. Group comparisons (n = 6 animals each group) were performed with either t-test or Mann-Whitney-U test. n.s.: not significant.
(TIFF)

S1 Table Full list of 41 differentially expressed genes in tumor tissue. (XLSX)

S1 Raw Data (XLSX)
