# Supplementary figures and images for: Sevoflurane depletes macrophages from the melanoma microenvironment
Source: PLoS One. 2020 May 29;15(5):e0233789. doi: 10.1371/journal.pone.0233789 (PMC7259700; doi:10.1371/journal.pone.0233789)

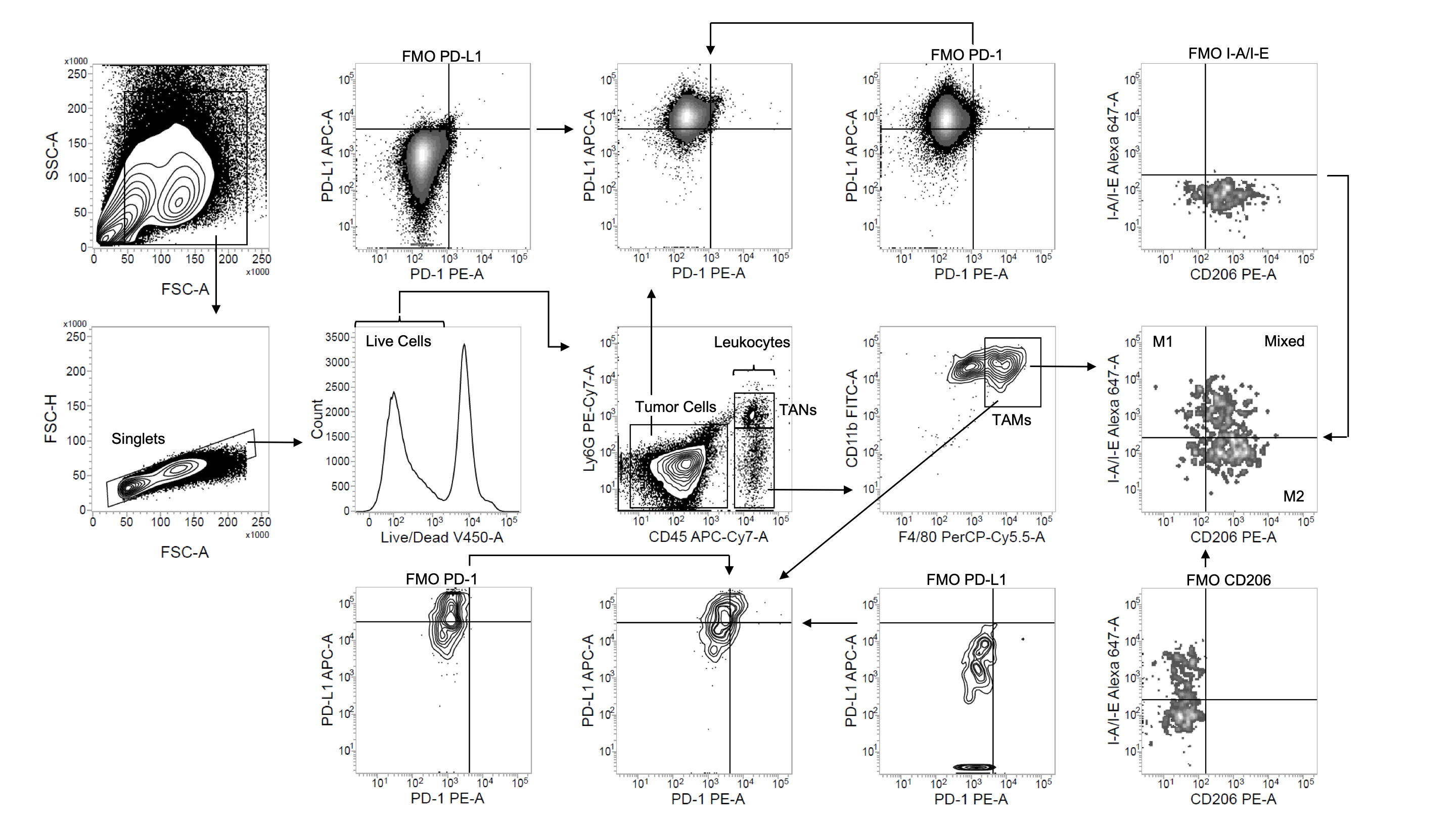

Supplement: S1 Fig — Gates were set according to fluorescence-minus-one (FMO) controls as indicated in the example (plots with “FMO” in headline). (TIFF) [file pone.0233789.s001.tiff]

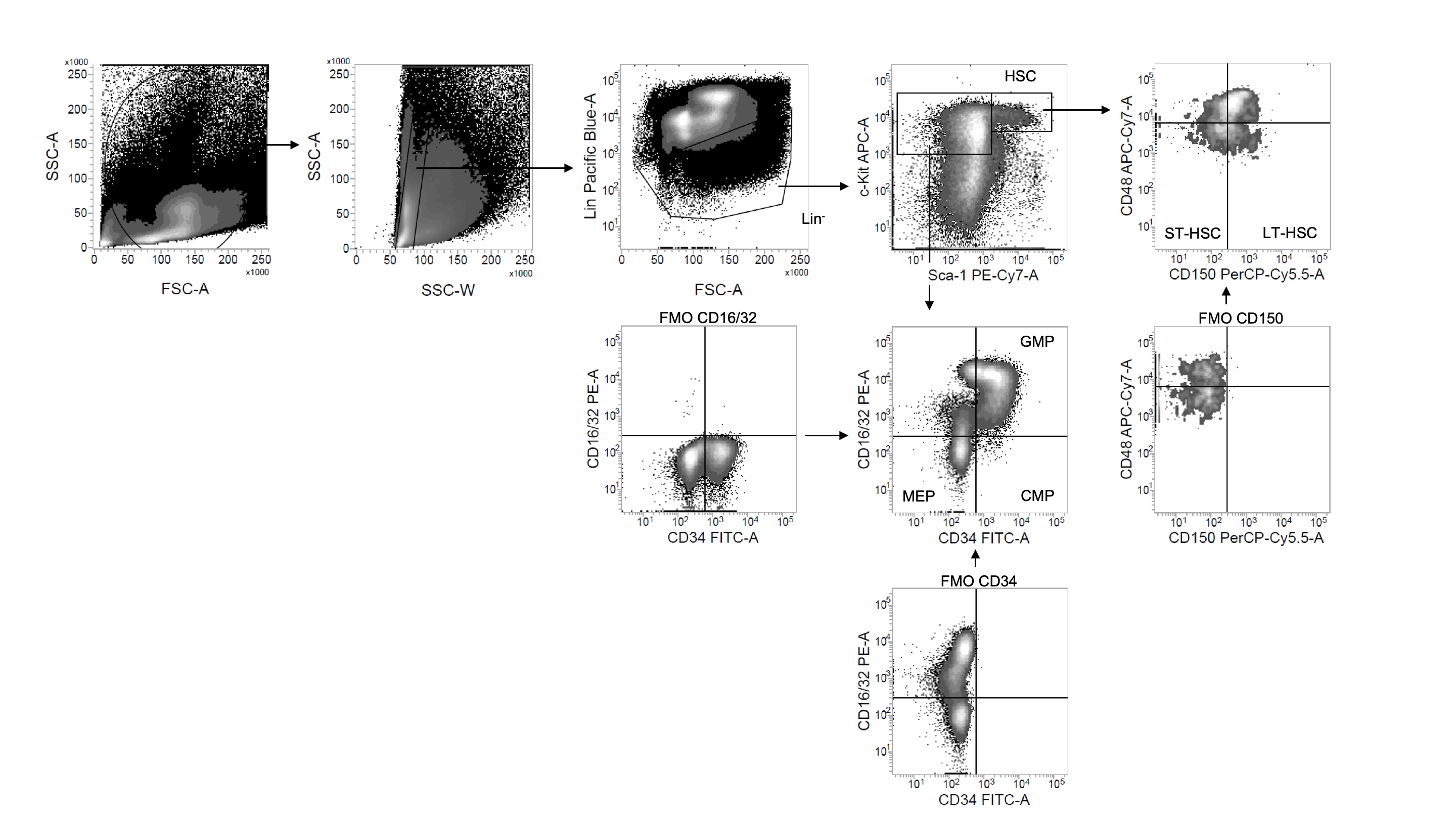

Supplement: S2 Fig — Gates were adjusted according to fluorescence-minus-one (FMO) controls as indicated in the example (plots with “FMO” in headline) where necessary. HSC: Hematopoietic stem cells; LT-HSC: Long-term-HSC; ST-HSC: Short-term-HSC; CMP: Common myeloid progenitor; MEP: Megakaryocyte-erythroid progenitor; GMP: Granulocyte-monocyte progenitor. (TIFF) [file pone.0233789.s002.tiff]

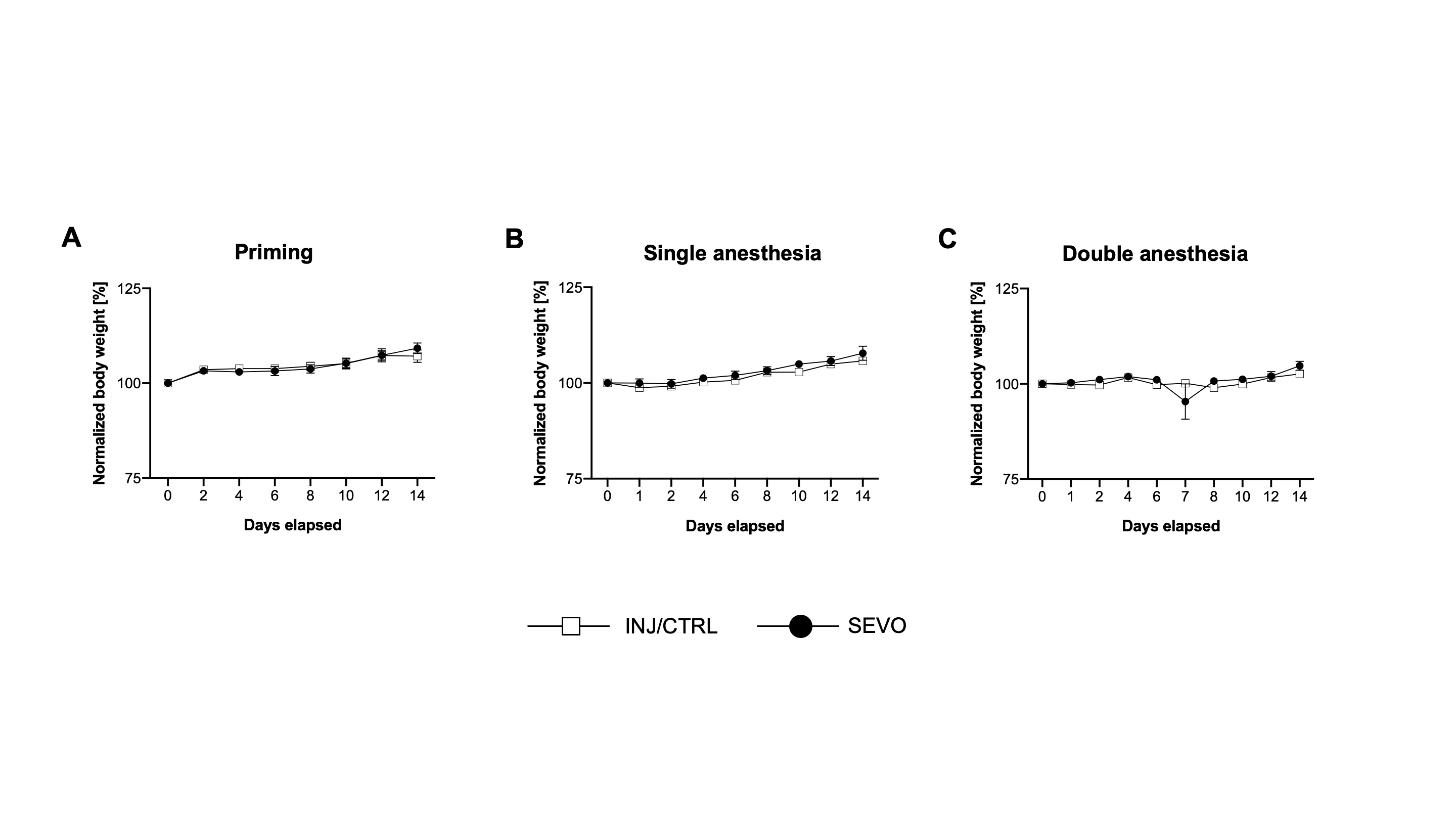

Supplement: S3 Fig — Mice receiving (A) implantation of primed tumour cells (n = 8 animals each group), (B) single anesthesia (n = 7 animals each group), or (C) double anesthesia (n = 7 animals each group). INJ: group receiving injection anesthesia (white boxes); CTRL: group receiving non-primed B16-F10 cell (white boxes); SEVO: groups receiving sevoflurane anesthesia or sevoflurane-primed B16-F10 cells, respectively (black circles). (TIFF) [file pone.0233789.s003.tiff]

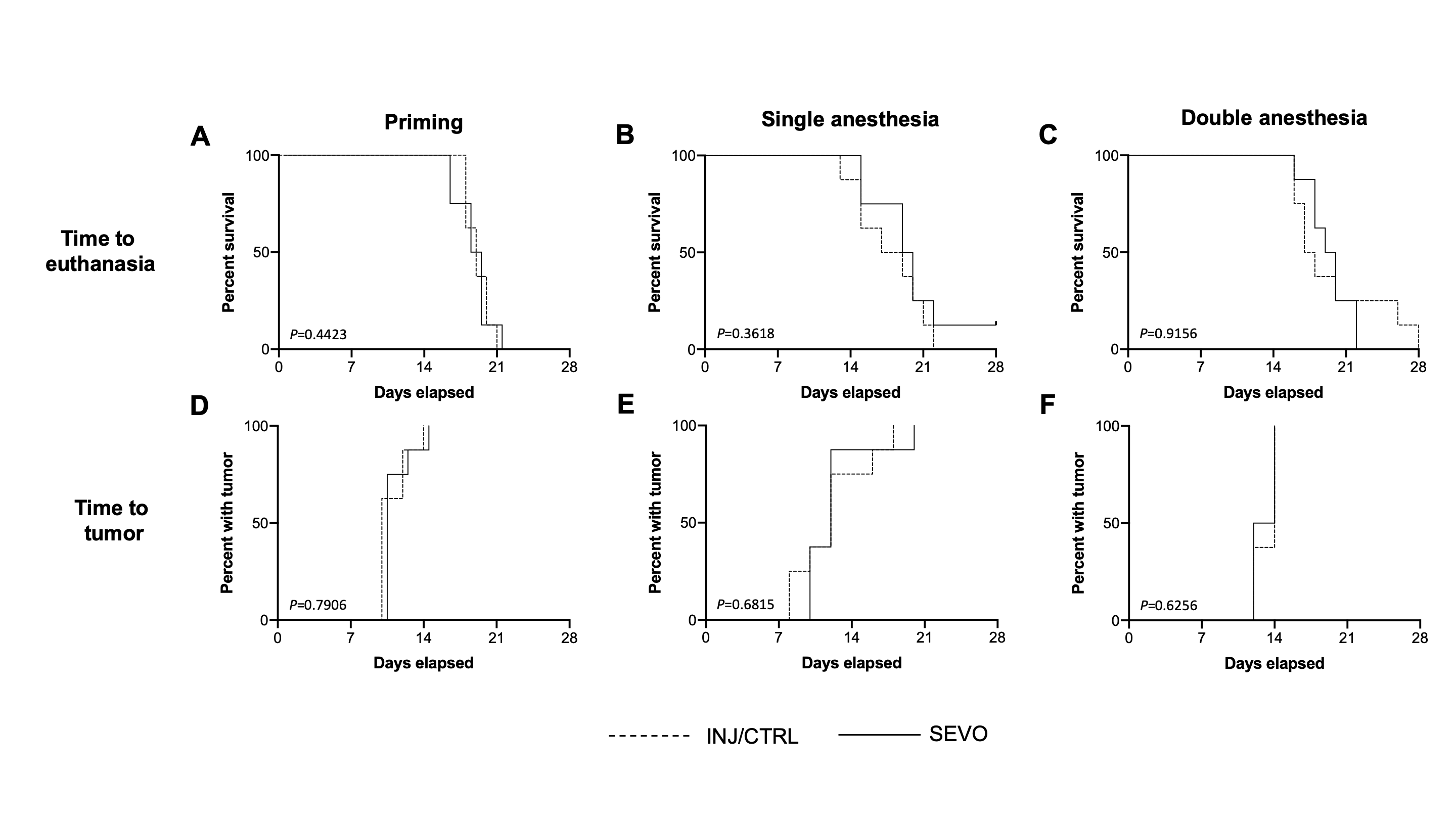

Supplement: S4 Fig — Kaplan-Maier curves of time to euthanasia (A-C) or time to palpable tumour (D-E). Animals received primed B16-F10 cells (A+D), a single anesthesia (B+E), or double anesthesia (C+F). Each group with n = 7 animals with exception of both subgroups of the priming experiment, which has n = 8 animals in each group. P-values represent results of Log-rank test. INJ: group receiving injection anesthesia; CTRL: group receiving non-primed B16-F10 cell; SEVO: groups receiving sevoflurane anesthesia or sevoflurane-primed B16-F10 cells, respectively. (TIFF) [file pone.0233789.s004.tiff]

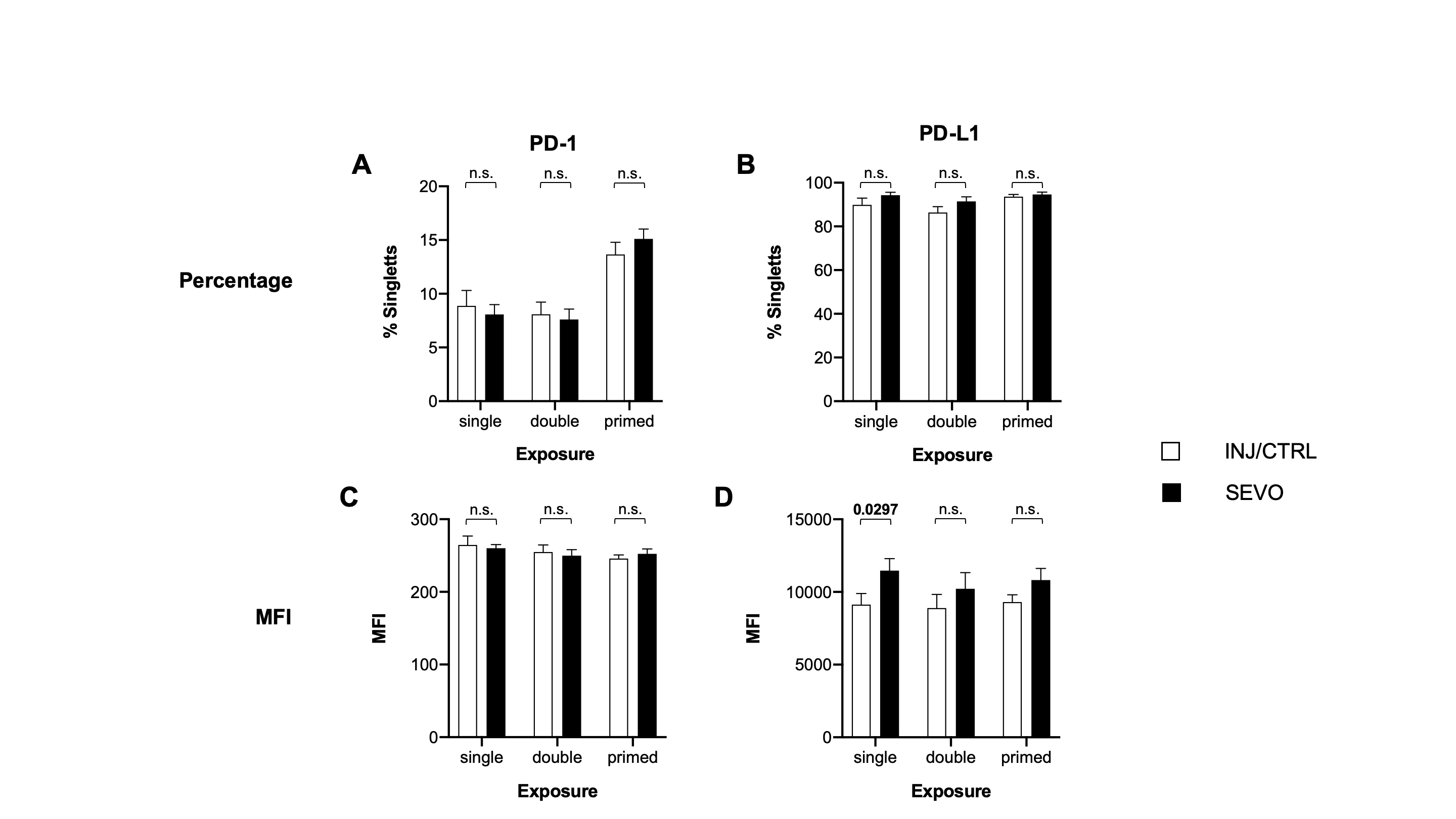

Supplement: S5 Fig — Expression of PD-1 (A+C) and PD-L1 (B+D) on tumour cells. Results are given either as fraction of positive singlet cells (A+B) or mean fluorescence intensity (MFI) (C+D). All experimental groups are combined within each graph, with “single and “double” indicating the number of anesthesia cycles received (n = 7 animals each group), and “primed” the group receiving primed B16-F10 cells or control cells, respectively (n = 8 animals each group). PD-L1: Programmed death-ligand 1, PD-1: Programmed death 1, TAN: tumour-associated neutrophils, MFI: mean fluorescence intensity, n.s.: not significant. Bars represent mean and standard error of mean, with white bars representing the control groups and black bars the sevoflurane groups. Bold number indicates significant difference (P-value<0.05) between groups, calculated with either t-test or Mann-Whitney-U test. (TIFF) [file pone.0233789.s005.tiff]

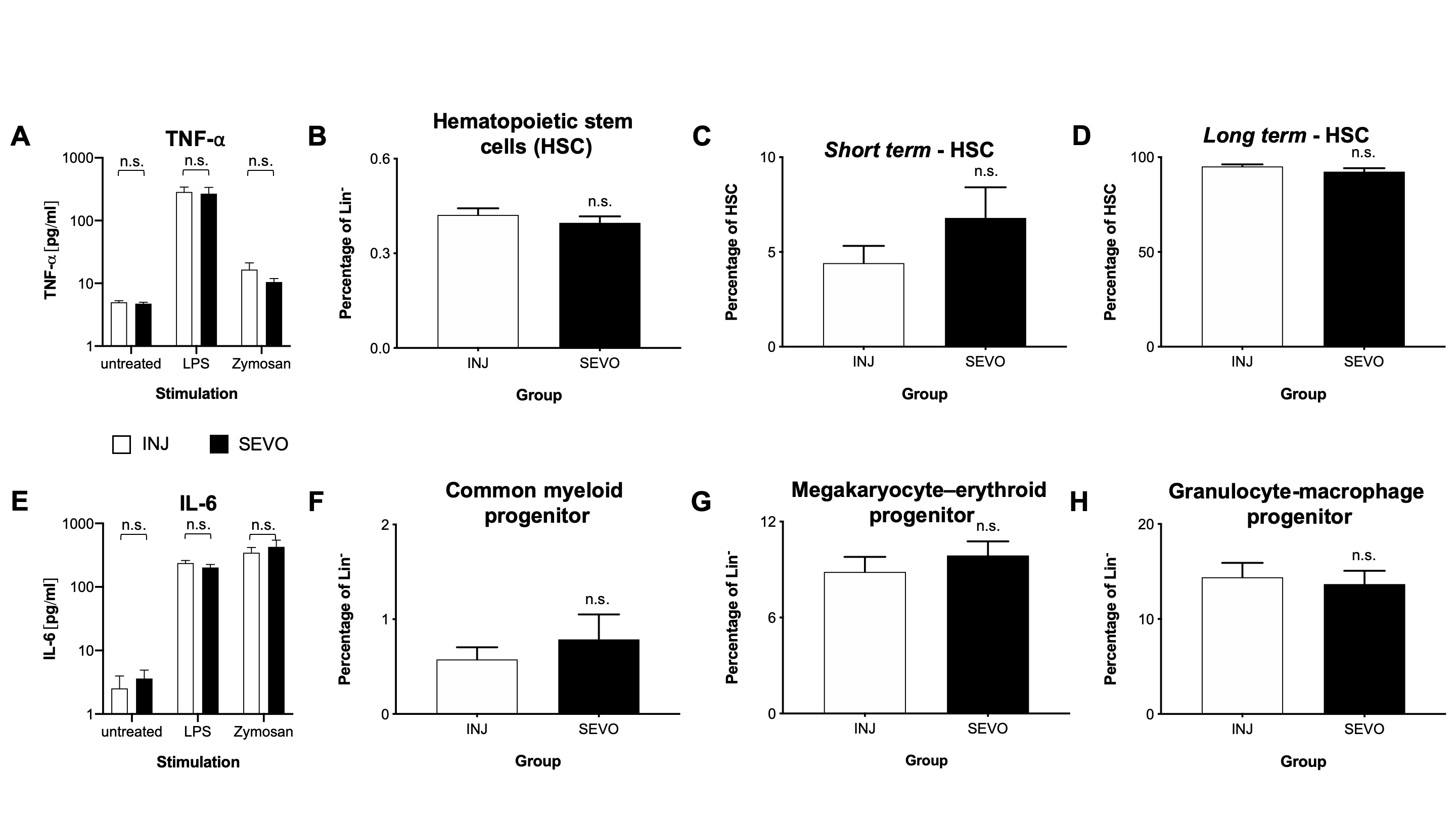

Supplement: S6 Fig — (A) TNF-α concentration (measured by ELISA) in the supernatant of bone marrow monocytes after 24h stimulation with 100ng/ml LPS or 250μg/ml zymosan (or untreated as control). White bars represent results of animals receiving injection anesthesia, black bars represent results from animals receiving sevoflurane anesthesia. Percentage of (B) hematopoietic stem cells (HSC) (C) Short-term HSC, and (D) Long term HSC. (E) IL-6 concentration (measured by ELISA) in the supernatant of bone marrow monocytes after 24h stimulation with 100ng/ml LPS or 250μg/ml zymosan (or untreated as control). Percentage of (F) Common myeloid progenitor, (G) megakaryocyte-erythroid progenitor, and (H) granulocyte-macrophage progenitor. Bars represent mean and standard error of mean. Group comparisons (n = 6 animals each group) were performed with either t-test or Mann-Whitney-U test. n.s.: not significant. (TIFF) [file pone.0233789.s006.tiff]
